# Nucleotide-binding oligomerization domain-containing protein 2 prompts potent inflammatory stimuli during *Neospora caninum* infection

**DOI:** 10.1038/srep29289

**Published:** 2016-07-05

**Authors:** Marcela Davoli-Ferreira, Denise M. Fonseca, Caroline M. Mota, Murilo S. Dias, Djalma S. Lima-Junior, Murilo V. da Silva, Gustavo F. S. Quirino, Dario S. Zamboni, João S. Silva, Tiago W. P. Mineo

**Affiliations:** 1 Laboratory of Immunoparasitology “Dr. Mário Endsfeldz Camargo”, Institute of Biomedical Sciences, Federal University of Uberlândia, Uberlândia, Minas Gerais, Brazil; 2 Department of Biochemistry and Immunology, Ribeirão Preto, School of Medicine, University of São Paulo, Ribeirão Preto, São Paulo, Brazil; 3 Program in Barrier Immunity and Repair, Mucosal Immunology Section, Laboratory of Parasitic Diseases, National Institute of Allergy and Infectious Disease (NIAID), National Institutes of Health (NIH), Bethesda, MD, USA; 4 Department of Cell Biology, Ribeirão Preto, School of Medicine, University of São Paulo, Ribeirão Preto, São Paulo, Brazil

## Abstract

*Neospora caninum* is an apicomplexan parasite responsible for major economic losses due to abortions in cattle. Innate immune responses are crucial for host resistance against the infection, however the molecules involved in parasite recognition are still poorly understood. Nod2 is a cytosolic receptor that recognizes several pathogens and its role during *N. caninum* infection has not yet been described. In that sense, we evaluated the role of Nod2 in host response against this parasite. We found that infection of macrophages induced increased expression of Nod2, which colocalized with the parasites’ vacuoles. Nod2-deficient macrophages showed an impaired induction of pro-inflammatory cytokines, increased production of modulatory molecules, and failure to restrict parasite replication. *In vivo*, Nod2-knockout mice showed a reduction of MAPK phosphorylation and proinflammatory cytokines, followed by decreased inflammation in target organs and increment in parasite burden. Surprisingly, these mice were partially resistant to lethal doses of tachyzoites. In addition, these phenomena were not observed in Rip2−/− mice. In conclusion, our study indicates that Nod2-dependent responses account for *N. caninum* elimination. On the other hand, the inflammatory milieu induced by this innate receptor provoked pathogenesis and death in severe experimental neosporosis.

*N. caninum* is an intracellular apicomplexan parasite capable of infecting a wide range of domestic and wild animals^1^,^2^,^3^. This parasite is the etiological agent of neosporosis, a severe infectious illness responsible for neuromuscular disorders in canines and abortion, neonatal mortality and congenital infections, imposing a high economic burden in cattle raising farms and associated industry^1^,^4^,^5^. Worldwide economic loss due to disease is estimated to up to US$2.3 billionannually^1^,^4^,^6^.

*N. caninum* presents three known infectious stages: fast replicating tachyzoites, bradyzoites present inside tissue cysts, and sporozoites within oocysts. To this date, the pathogenesis of neosporosis is associated with the intracellular proliferation of *N. caninum* tachyzoites. These parasite forms are disseminated through the blood stream and lymphatic system and can induce robust humoral and cellular immune responses, which are crucial to determine the development and severity of the disease^5^,^7^. Innate immunity plays an important role in protection of the host against protozoal infections, exerting essential role in the inhibition of initial parasite replication and the establishment of an appropriate adaptive immune response, in order to control active infections and consequently overcome re-exposures^8^,^9^.

Macrophages recognize and distinguish pathogens by the means of pattern recognition receptors (PRRs), a crucial mechanism for early parasite clearance, since it enables the host immune system to mount an appropriate inflammatory response. The best characterized PRRs are the *Toll*-like receptors (TLRs), which are transmembrane proteins localized either at the cell surface or within the endosome^10^,^11^. Previous studies have demonstrated that initial *N. caninum* recognition occurs by TLR2 and TLR3^12^,^13^, and engagement of these receptors trigger the activation of adaptor molecules Myd88 or TRIF and its respective pathways, directing the immune response against this parasite^8^,^12^,^13^.

In addition to the TLRs, Nod-like receptors (NLRs) have emerged as important components of the innate immune system, accounting for the detection of intracellular pathogens^14^,^15^. Nod1 and Nod2 are well-characterized members of the NLRs^16^,^17^. These receptors are localized in the cytoplasm and recognize pathogens that gain access to the host cell compartment, through lysis of the parasitophorous vacuole or injection of effector proteins through its membranes. While Nod1 is ubiquitously expressed, Nod2 is expressed in hematopoietic cells, and both cooperate to signal via the adaptor molecule Receptor-Interacting Protein 2 (Rip2), a serine-threonine kinase required for activation of NF-κB and mitogen-activated protein kinase (MAPK) cascades that culminate with the upregulation of proinflammatory genes^18^,^19^,^20^. Nod2 is involved in the recognition of active moieties of bacterial cell wall peptidoglycan, muramyl dipeptide (MDP), and previous studies state that this receptor may be crucial to host defense against infections by parasites and viruses^19^,^21^,^22^,^23^,^24^. Also, it has been proposed that Nod2 is necessary for generating protective Th1 immunity against *T. gondii,* a protozoan parasite closely related with *N. caninum*^22^.

Since Nod2 contributes to development of innate immune responses against several pathogens, including protozoan parasites, we investigated if Nod2 participates in the innate immune recognition and host resistance against *N. caninum*. Strikingly, we observed that despite decreased pro-inflammatory cytokine production and increased of burden parasite, Nod2^−/−^ mice were highly resistant to lethal infections. Taken together, our data indicate that Nod2 activation during *N. caninum* infection exacerbates the inflammatory response, contributing to initial parasite control, although inducing pathogenesis and consequent increased susceptibility during acute neosporosis.

## Results

### Nod2 is involved in macrophage responsiveness against *N. caninum* infection

**Figure 1:**
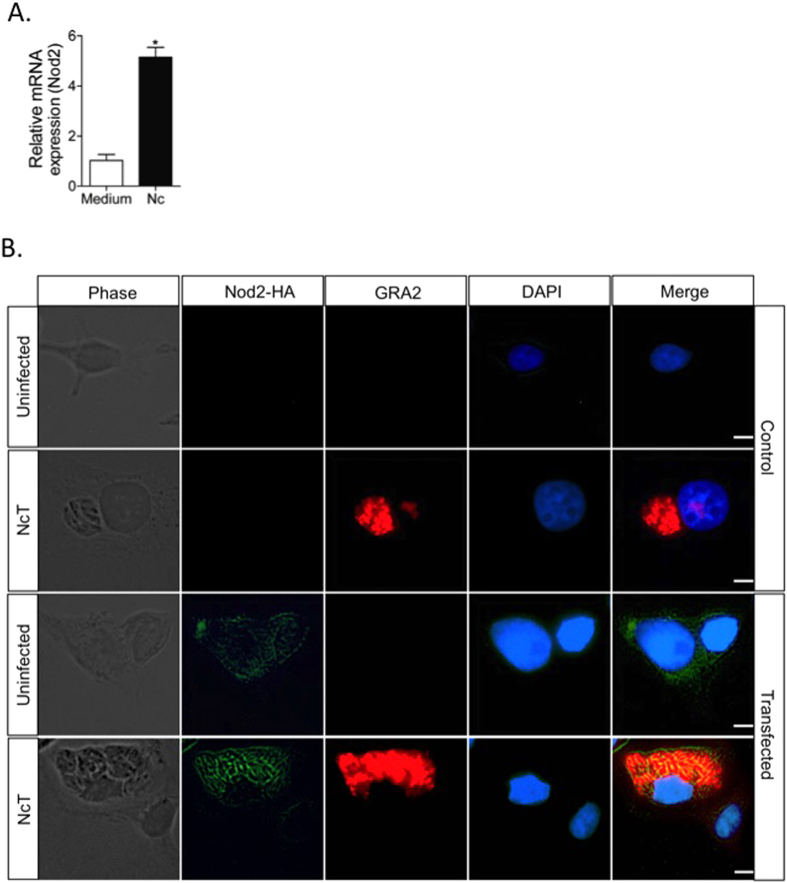
Nod2 is expressed and recruited to the vacuole during *N. caninum* infection. The expression of Nod2 following infection of WT BMDMs with *N. caninum* tachyzoites (MOI 0.2) was evaluated 6 h post-infection by Real time PCR (**A**). Results were represented as relative expression of the target gene, with CT data normalized by the expression of *Gapdh* and are shown as means of biological triplicates and the data are representative of two independent experiments. *Statistical difference (P ≤ 0.05) between naïve and *N. caninum* infected WT BMDMs. Nod2-HA-transfected HEK293 cells were infected with *N. caninum* tachyzoites (NcT; MOI 1) and the recruitment of this receptor to the parasitophorous vacuole was evaluated by fluorescence microscopy 24 h post-infection (**B**). Besides Nod2-HA detection (green), uninfected and NcT exposed, control HEK293 and HEK293-Nod2-HA+ cells were also stained with a monoclonal antibody against the secreted GRA2 protein of *N. caninum* (red) and DAPI (blue). The original images were obtained with 400x magnification (white scales bars = 10 μm).

To assess the pattern of Nod2 expression during the infection of macrophages, we evaluated the messenger RNA codifying this receptor in mouse bone marrow derived macrophages (BMDMs) during *N. caninum* infection *in vitro*. For this purpose, BMDMs from C57BL/6 mice were infected with *N. caninum* tachyzoites (MOI 0.2) and analyzed for Nod2 transcript levels. After 6 hours of infection, we observed a significant increase (P < 0.05) in Nod2 relative transcript expression in BMDMs exposed to live tachyzoites (Fig. 1A).

In order to observe whether the expression pattern of this receptor was altered by the infection, we observed Nod2 protein expression pattern by microscopy. For that experiment, we used HEK293 cells transfected in order to overexpress Nod2 receptors fused to a HA tag. With that experimental layout, we observed that Nod2 was directly recruited to the parasite vacuole, evidenced by the colocalization of parasite excreted GRA2 protein, as the Nod2-HA constructs co-localized with individual tachyzoites (MOI 1; Fig. 1B), indicating that this innate receptor is activated during *N. caninum* infection.

**Figure 2:**
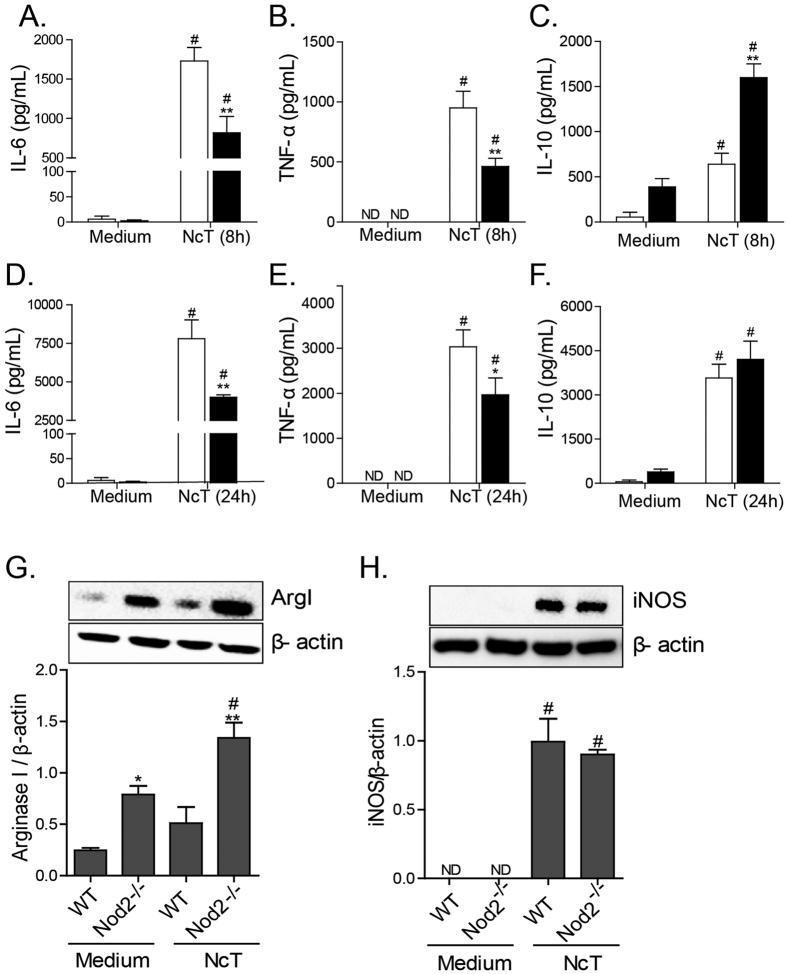
Nod2 leads to pro-inflammatory responses in macrophages during *N. caninum* infection. The levels of IL-6 (**A,D**), TNF-α (**B,E**) and IL-10 (**C,F**) were measured by ELISA in cell-free supernatants of naïve WT and Nod2^−/−^ BMDMs, or infected with *N. caninum* tachyzoites (MOI 0.2), at 8 h (**A–C**) and 24 h (**D–F**) post-infection. Arginase I (**G**) and iNOS (**H**) expression were quantified, by Western Blot, in lysates of WT and Nod2^−/−^ BMDMs, 48 hours post-infection with *N. caninum* tachyzoites (MOI 0.2). All results are shown as means of triplicate cultures of BMDMs and the data are representative of at least two independent experiments. Error bars indicate mean ± SEM. ^#^P ≤ 0.05 compared to the medium; *P ≤ 0.05, **P ≤ 0.01 compared to WT group. ND = not detected.

To determine whether Nod2 was related to triggering innate immune responses against live *N. caninum* tachyzoites, macrophages were infected with tachyzoites (MOI 0.2) for cytokine measurement after 8 h and 24 h. Infection of WT macrophages with *N. caninum* induced a marked production of IL-6 (Fig. 2A,D) and TNF-α (Fig. 2B,E), and this response was partially suppressed in macrophages lacking Nod2, whereas IL-10 levels were increased in culture supernatants of Nod2^−/−^ BMDMs at the initial replicative cycle of the parasite (8 h, Fig. 2C). Although WT and Nod2^−/−^ BMDMs expressed similar amounts of inducible nitric oxide synthase (iNOS, NOS2) after co-culture with live tachyzoites (Fig. 2H), infected Nod2^−/−^ cells expressed significantly higher levels of Arginase I (ArgI, P < 0.01, Fig. 2G), an antagonist of nitric oxide production, which in turn is an important effector molecule produced by innate immune cells to control parasite replication.

Collectively, these results indicate that Nod2 is recruited to the vacuole during the infection, contributing to the production of pro-inflammatory cytokines and nitric oxide.

### Nod2-dependent responses contribute with parasite clearance

**Figure 3:**
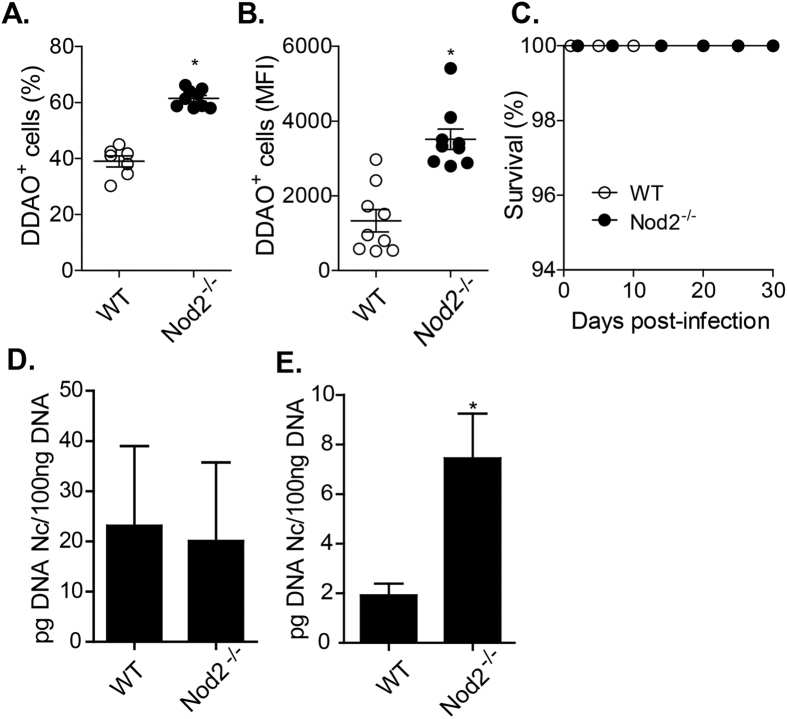
Nod2-dependent response is required for the restriction *of Neospora caninum* replication. BMDMs from WT and Nod2^−/−^ mice were infected with DDAO-stained *N. caninum* tachyzoites (MOI 0.2) and evaluated for the percentage of infected cells (**A**) and the mean fluorescence (**B**), after 24 h of infection, by flow cytometry. Results are shown as the mean of nine biological replicates and are representative of two independent experiments. Nod2^−/−^ and WT mice were infected with sub-lethal doses of *N. caninum* tachyzoites (1.10^7^ tachyzoites, i.p. route) and the survival was evaluated for 30 days (*n* = 8 mice per group, (**C**)). In a parallel experiment, peritoneal exudate cells (**D**) and pancreas (**E**) of mice submitted to sub-lethal infections were checked for parasite load by quantitative PCR after 5 days of infection (*n* = 5 mice per group). *Statistical difference (*P ≤ 0.05) between WT and Nod2^−/−^ mice.

Since Nod2 induced the production of pro-inflammatory mediators by BMDMs, while dampening IL-10 production, we then opted to investigate if the absence of Nod2 would influence the ability of the host to restrict *N. caninum* replication. We found that Nod2^−/−^ BMDMs presented a higher proportion of infected cells after 24 h of infection (Figs 3A and S2), in addition to an increased median intensity of fluorescence (MFI; Fig. 3B), suggesting that Nod2^−/−^ BMDMs are susceptible to invasion and parasite replication, if compared to WT cells. To assess whether Nod2 regulates the clearance parasite *in vivo*, WT and Nod2^−/−^ mice were infected with sub-lethal doses of tachyzoites (Fig. 3C), and the parasite burden was evaluated after 5 days of infection in peritoneal exudate cells (Fig. 3D) and pancreas (Fig. 3E). Although the absence of functional Nod2 did not compromise the survival and parasite clearance at the initial site of the infection, Nod2^−/−^ mice presented a four-fold higher parasite load in the pancreas (Fig. 3E), if compared to infected WT littermates. Together, these data suggest that Nod2-triggered responses are required, although not essential, for *N. caninum* growth restriction during *in vivo* infection.

### Nod2 induces aggravated inflammation and lethality during *N. caninum* infection

**Figure 4:**
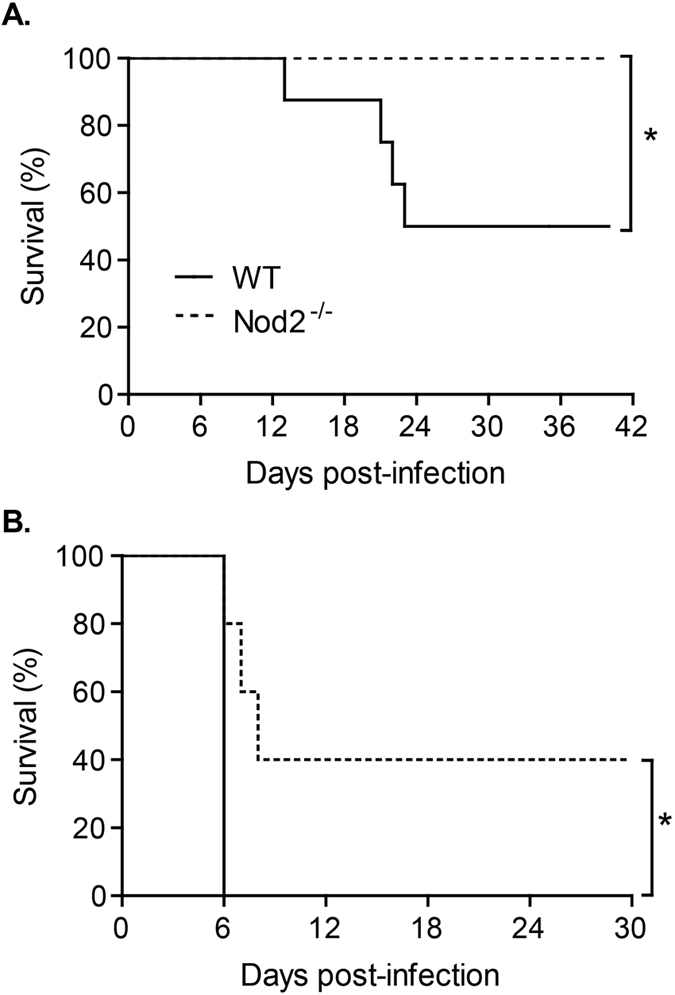
The presence of functional Nod2 increases lethality induced by *N. caninum* infection. Nod2^−/−^ and WT mice were infected with different doses of *N. caninum* tachyzoites, able to kill ~50% (LD50, n = 8 mice per group; (**A**)) or 100% (LD100, n = 5 mice per group; (**B**)) of the mice and the survival was evaluated for 40 and 30 days, respectively. Differences in survival rates between groups were compared using Kaplan-Meier survival analysis, through a log-rank Mantel-Cox test. Data are representative of three independent experiments. *Statistical difference (*P ≤ 0.05) between WT and Nod2^−/−^ mice.

To assess whether Nod2 is linked with susceptibility or resistance to the infection, Nod2^−/−^ mice were infected with doses of *N. caninum* tachyzoites lethal to ~50% (LD50; Fig. 4A) or ~100% (LD100; Fig. 4B) of the WT mice. As expected, 50% of WT mice succumbed to a LD50 infection and, surprisingly, no deaths recorded in Nod2^−/−^ group (Fig. 4A). The reduced mortality of Nod2^−/−^ mice was also observed after LD100 infection protocols; approximately 40% of these mice survived during the observation period of 30 days (Fig. 4B).

**Figure 5:**
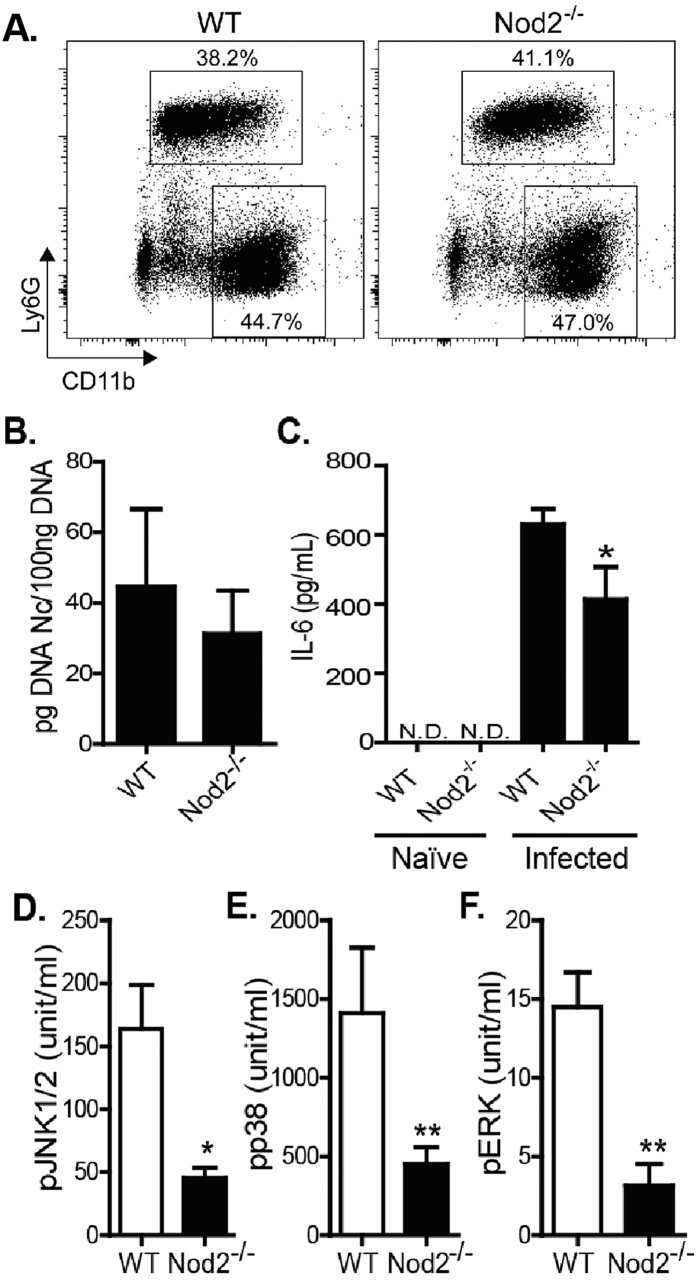
Nod2 mediate MAPK phosphorylation and proinflammatory cytokine production during *in vivo* infection. Peritoneal exudates cells from WT and Nod2^−/−^ mice infected with LD100 were collected and the influx of CD11b^+^ (macrophages) and Ly6G^+^ cells (neutrophils) was evaluated by flow cytometry (**A**). Representative percentages of the populations are shown beside each gate. Parasite burden was analyzed in the peritoneal exudates cells by Real Time PCR (**B**) and IL-6 (**C**) levels were measured in peritoneal exudates by ELISA (n = 4–5 mice per group). The levels of pJNK1/2 (**D**), pp38 (**E**), pERK (**F**) were quantified in peritoneal cell lysate, by Cytometry Bead Array (n = 4 mice per group). *Statistical difference (*P ≤ 0.05; **P ≤ 0.01) between WT and Nod2^−/−^ mice.

Given that Nod2^−/−^ mice showed increased resistance to infection, despite the lack of adequate control of parasite loads in determined tissues, we speculated if these phenomena could be associated with a reduction of tissue damage due to a milder inflammatory response. In an initial experiment, we evaluated the influx of inflammatory cells to the site of infection (peritoneal cavity) after a LD100 infection. We observed that the absence of functional Nod2 did not alter the absolute number of peritoneal exudates cells after 5 days of infection (data not shown), as well as the proportion of macrophages (CD11b^+^ cells), neutrophils (Ly6G^+^ cells) and CD4^+^ cells (Figs 5A and S1). Moreover, confirming the result obtained with sub-lethal tachyzoite infections, no differences were noted in the parasite loads of peritoneal cells of WT and Nod2^−/−^ mice (Fig. 5B). On the other hand, we detected a significant reduction in IL-6 concentration on the peritoneal exudates of genetically deficient mice (Fig. 5C) after 5 days of infection. Since Nod2 has been pointed to activate downstream MAPK pathways, which in turn are responsible for pro-inflammatory cytokine production and effector responses, we assessed whether the absence of the receptor compromised the phosphorylation of these molecules in cells at the initial site of infection during lethal infection with *N. caninum* tachyzoites. Strikingly, Nod2 genetic depletion induced a three-fold reduction (P < 0.05) in the phosphorylation of JNK1/2 (Fig. 5D), p38 (Fig. 5E) and ERK (Fig. 5F) of peritoneal cells obtained from mice under LD100 infection (Representative dot plots in Fig. S3).

**Figure 6:**
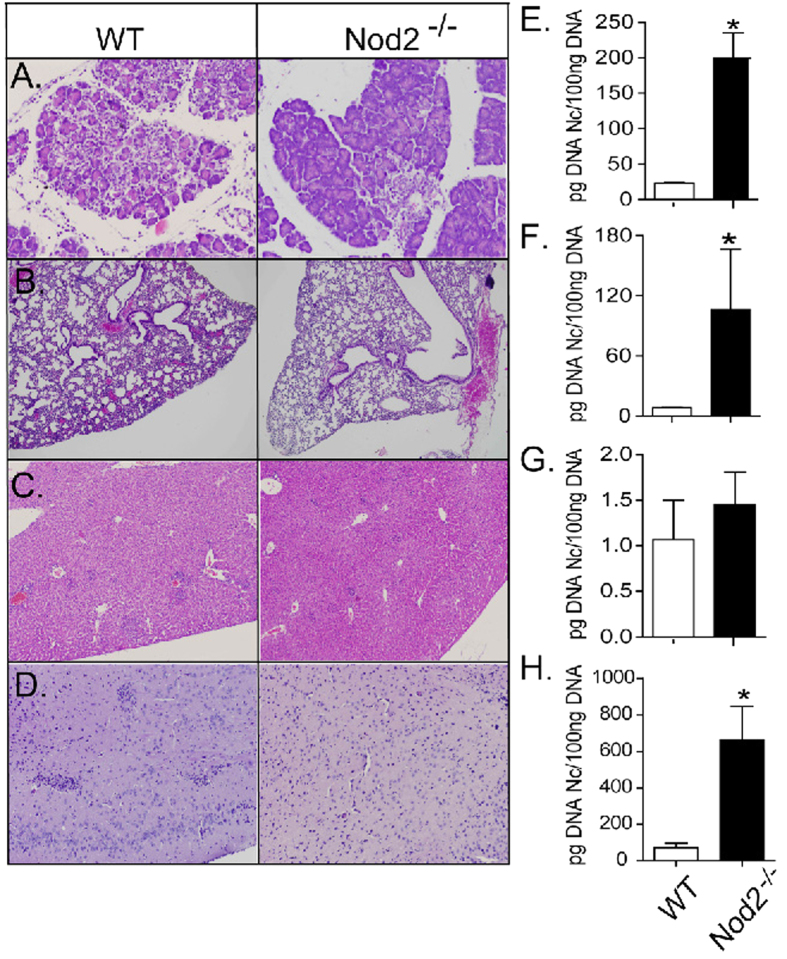
Nod2 upregulates inflammatory responses while controls *in vivo N. caninum* proliferation in target tissues. Representative histological images of the pancreas ((**A**), 150×), lungs ((**B**), 40×), liver ((**C**), 60×) and brain ((**D**), 100×) obtained from WT and Nod2^−/−^ mice, 5 days after infection (LD100). Parasite loads were evaluated in pancreas (**E**), lungs (**F**), liver (**G**) and brain (**H**) samples by Real Time PCR (*n* = 5 mice per group). Data are representative of two independent experiments. *Statistical difference (*P ≤ 0.05) between WT and Nod2^−/−^ mice.

Although no differences were previously detected in the influx of immune cells to the initial site of the infection, we evaluated the inflammatory influx to other tissues targeted by the acute parasite dissemination. We observed that, 5 days after the infection with lethal tachyzoite doses, pancreas from WT mice showed large areas of edema, as well as diffused inflammatory infiltrates composed mainly by mononuclear cells, clearly compromising organ integrity (Fig. 6A). Notably, these inflammatory indicators were markedly attenuated in the pancreas of infected Nod2^−/−^ mice (Fig. 6A). We also observed that Nod2^−/−^ mice also presented decreased thickening of the lung parenchyma and consequent loss of alveolar area during lethal challenge, if compared to WT mice (Fig. 6B). No apparent differences were observed between the groups regarding the inflammatory profile of hepatic tissues (Fig. 6C). Moreover, samples obtained in the same experiments revealed the formation of perivascular cuffs and focal inflammatory infiltrates composed mainly by mononuclear cells in the brain parenchyma of WT mice, conditions that were present, although in a milder fashion, in samples obtained from Nod2^−/−^ mice (Fig. 6D). Furthermore, the concentration of parasite genomic DNA was significantly increased (P ≤ 0.05) in the tissues analyzed, with the exception of liver samples (Fig. 6E–H).

These results indicate that Nod2 contributes to the restriction of parasite replication during the acute lethal infection. However, this control is based on a significant upregulation of inflammatory hallmarks in targeted organs, which may compromise tissue integrity and, consequently, host survival.

### Nod2 signaling during *N. caninum* infection is independent of Rip2

**Figure 7:**
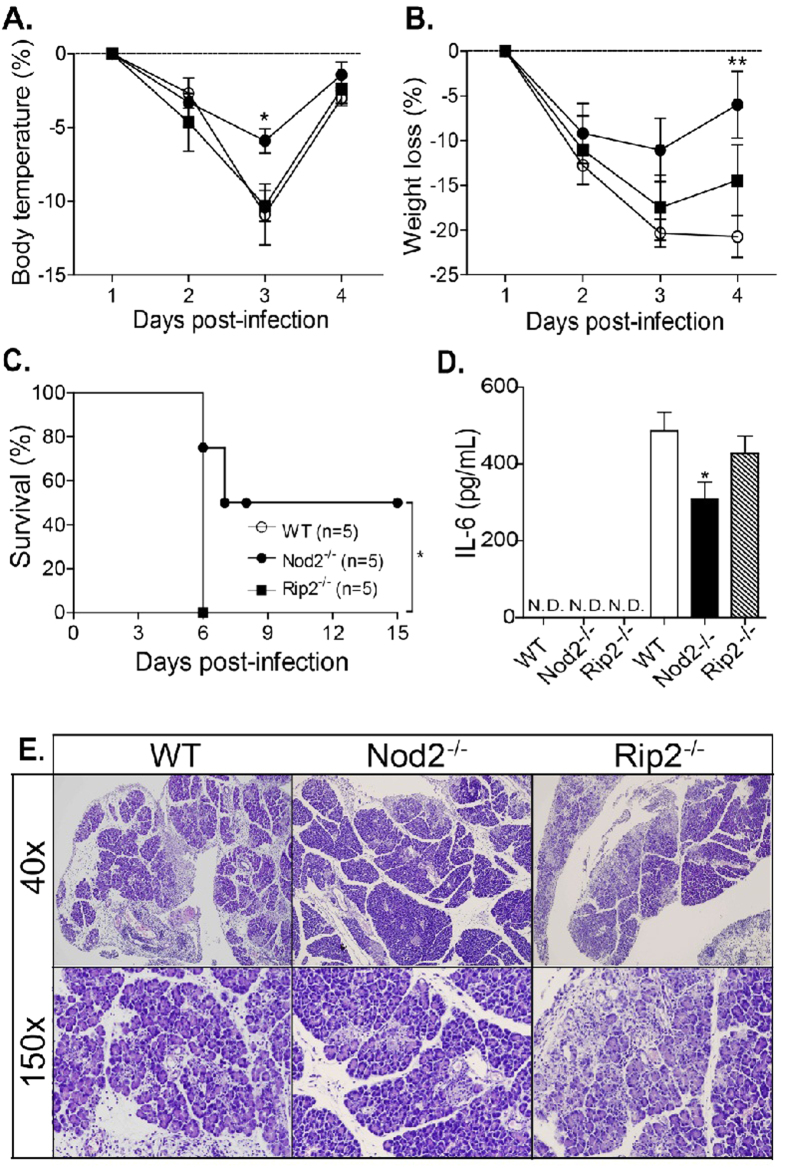
Nod2-induced pathogenesis during lethal infections is independent of adaptor protein Rip2. Nod2^−/−^, Rip2^−/−^ and WT mice (n = 5 mice per group) were infected with *N. caninum* tachyzoites (LD100) and the body temperature (**A**), weight (**B**) and survival (**C**) of these mice were monitored over time. Statistical significance of the parameters was calculated using two-way analysis of variance followed by Bonferroni post-hoc test. Survival curves were compared using Kaplan–Meier survival analysis through log-rank Mantel–Cox test. In another experimental group, Nod2^−/−^, Rip2^−/−^ and WT mice (n = 5 mice/group) were infected with sub-lethal doses of tachyzoites (1.10^7^ tachyzoites) and the levels of IL-6 were measured in peritoneal exudates by ELISA, 5 days post-infection (**D**). Histological analysis was realized in pancreas (**E**) collected from the groups. *Statistical difference (*P ≤ 0.05; **P ≤ 0.01) between WT mice and the deficient mouse lineages tested.

Since Rip2 is the best-studied interaction partner of Nod2, we evaluated whether this adaptor protein was involved in Nod2 triggered phenomena during the infection. Rip2^−/−^ mice infected with lethal doses of *N. caninum* tachyzoites exhibited decrease in body temperature (Fig. 7A), severe weight loss (Fig. 7B) and succumbed to infection (Fig. 7C), in a similar manner to that observed in the WT mice group. Additionally, Rip2^−/−^ mice infected with sub-lethal doses of tachyzoites presented an inflammatory response similar to WT mice. In the absence of Rip2, the concentration of IL-6 in the peritoneal exudates (Fig. 7D) and the inflammatory response in the pancreas (Fig. 7E) were similar to the response observed in WT mice, with large edema areas and an intense and diffused inflammatory infiltrate, seriously compromising tissue integrity. Taken together, these data indicate that the Nod2-mediated inflammation induced during *N. caninum* infection is induced through a Rip2-independent mechanism.

## Discussion

Innate immune recognition of intracellular pathogens is crucial for the generation of effector immune responses that culminates in the restriction of microbial multiplication and, in most cases, in resistance to infection. Although protozoan infections are extremely relevant to the Veterinary field, little information is available on the innate signaling pathways triggered by these organisms, in contrast to the broad literature on innate responses to viral and bacterial pathogens^13^. The major aim of our study was to determine if the cytosolic receptor Nod2 was associated with innate immune responses developed during *N. caninum* infection. Up to date, there is no description of a role of NLRs during infection with this protozoan parasite. Here, we show that Nod2 is overexpressed and recruited towards the parasitophorous vacuole, triggering MAPK activation, proinflammatory cytokine production and parasite clearance *in vitro* and *in vivo*. In fact, it has been demonstrated that Nod2 can be directed to the intracellular vesicle compartment and cell surface membranes after muramyl-dipeptide (MDP) stimulation, and that this membrane association of Nod2 is necessary for NF-κB and MAPK activation^25^. However, due to the experimental approaches adopted in this study, it is still unclear how this recruitment is modulated and whether the parasite directly interferes with its functions.

Nod2 is classically activated by MDP, a conserved structure from bacterial peptidoglycan. Nevertheless, increasing numbers of reports suggest that it also has important functions during non-bacterial infections (as reviewed in 18 and 21). To our knowledge, there is no description of *N. caninum* agonists for Nod2. We speculate that *N. caninum* may trigger Nod2 by proteins injected into the host cytosol during the invasion and replication process, a common feature of the members of the Apicomplexa phylum^26^. We also find possible that Nod2 may recognize danger-associated molecular patterns (DAMPs) brought forth during the infection, since some studies have proposed that this innate receptor can sense these signals generated due to disruption of host cell membranes, a common event during tachyzoites egress^27^,^28^.

Our data provide evidence that Nod2 activation led to a complementary production of proinflammatory cytokines, thus, consequently, minimizing the risks of a deleterious reactivation of the infection. Our group has previously demonstrated that TLRs are essential for host resistance against neosporosis. *Myd88* and *Tlr2* ablation produced pronounced effects over the development of a specific Th1 response, based on the classical IL-12/IFN-γ axis, during the course of the infection. However, the TLR2-MyD88 signaling pathway partially controls IFN-γ production during the infection, suggesting that other PRRs may recognize *N. caninum* and participate in the effective immune responses^8^,^12^. In that sense, our results suggest that Nod2 is an alternative activation pathways that cooperate with TLRs in order to control *N. caninum* replication. This hypothesis is further corroborated by previous studies demonstrating that TLRs cooperate with Nod1 and Nod2 to control bacterial infections *in vivo*^29^. In this context, future studies with double deficient mice for TLRs and NLRs are desirable in order to elucidate the cooperation of innate immune sensors for the recognition of intracellular protozoa. On other hand, it is clear that the absence of Nod2 signaling leads to significantly increased production of anti-inflammatory molecules, such as IL-10 and arginase I, in macrophages infected with live parasites. Nod2 has a dual role in the expression of IL-10 during inflammatory processes. Some studies indicate that this receptor is crucial for the induction of IL-10 expression during fungal infection and in Crohn’s disease models. Meanwhile, high levels of this cytokine are produced in the absence of Nod2 during ehrlichiosis^29^,^30^,^31^. This result is also in agreement with our previous data showing that TLR2^−/−^ and MyD88^−/−^ mice presented increased IL-10 production after the infection^8^,^12^. The mechanisms underlying IL-10 production during *N. caninum* infection and the involvement of TLRs and Nod2 in inhibiting its expression are still unknown and should be further investigated.

Surprisingly, despite the increased parasite burden and the defective inflammatory response observed in *Nod2*-deficient mice, these animals showed increased survival when submitted to infection protocols with lethal doses of tachyzoites. This increment in resistance was associated with a milder inflammatory condition in key organs, with special emphasis on the pancreas. In that sense, we speculate that Nod2 is a substantial element in the pathogenesis of neosporosis, characterized mainly by an exacerbated inflammation that culminates in extensive tissue damage and lethality. The concept of tolerance to infections has been widely discussed in the last years and it speculated that it is more relevant to reduce the negative impacts of an infection than sterile immunity, which causes tissue damage and further host sickness (as reviewed in^32^ and^33^). In this scenario, we suggest that Nod2 activation by *N. caninum* triggers an increment in the inflammatory profile that may hamper the hosts’ tolerance after a parasite inoculum with high magnitude.

Additionally, our results suggest that pathogenesis induced by Nod2 after lethal infection protocols with *N. caninum* is independent of the canonical signaling pathway for this receptor. As previously described, Nod2 activation is mainly dependent on the recruitment and activation of Rip2, a serine-threonine kinase adapter molecule, essential for the activation of MAPK and NF-κB, resulting in the production of proinflammatory cytokines and chemokines^34^. In a distinct manner than that observed for Nod2, genetic depletion of Rip2 did not increase mice survival against lethal challenge, nor did alter IL-6 production and lesions observed in the pancreas. This finding, though unexpected, is consistent with some published results demonstrating that Nod2 can interact with others known innate pathways, such as those coordinated by mitochondrial antiviral signaling (MAVS), caspase activation and recruitment domain 9 (CARD9) and Ly6/PLAUR domain–containing protein 6 (LYPD6)^24^,^35^,^36^.

Altogether, the data herein gathered demonstrate that Nod2 is recruited to the parasitophorous vacuole and is activated during *N. caninum* infection, inducing the production of inflammatory mediators and consequent control of the parasite replication. However, we also observed that this receptor might be a key element of the pathogenesis of fatal infections by the parasite in a mouse model, which may be transposed to the clinical disease presented by puppies, contributing to the exacerbated inflammatory response and subsequent injuries of key tissues. Moreover, the role of Nod2 activation in abortions episodes in infected cattle should be verified, since the overproduction of proinflammatory cytokines may be also associated with placental damage leading to fetal death. Although complementary studies should be undertaken to characterize the exact mechanism of action of Nod2 during the infection, the results presented here highlights this intracellular receptor as a potential target for therapy against the clinical manifestations of the disease. We propose that treatment protocols with potential Nod2 antagonists, associated or not with other drugs, could reduce deaths and abortions caused by *N. caninum*.

## Methods

### Animals

All the experiments were developed in accordance to ethical principles in animal research adopted by Brazilian Society for Laboratory Animal Science and approved by the Ethics Committee on Animal Experiments, School of Medicine of Ribeirão Preto (process no. 195/2011). The proposed experiments were performed with female C57BL/6 wild type (WT), *Nod2*-deficient (Nod2^−/−^) and *Rip2*-deficient (Rip2^−/−^) mice, with 6–8 weeks of age. Deficient mice were kindly provided by Richard Flavell (Yale University). All mice were bred and maintained in small groups inside isolator cages, with light/dark cycle of 12 hours, food and water *ad libitum*, inside the animal housing facility of School of Medicine of Ribeirão Preto, University of São Paulo.

### Parasites and experimental infection

*N. caninum* tachyzoites (Nc-1 isolate) were propagated in monkey kidney cells (LLCMK2) in RPMI-1640 medium (Gibco-BRL, Gaithersburg, MD, USA), supplemented with 2 mM L-glutamine, 100 U penicillin, and 100 μg of streptomycin (all from Life Technologies, Carlsbad, CA, USA), in a 5% CO_2_ atmosphere, at 37 °C. Parasites were harvested after 80% lysis of host cell monolayer by mechanical disruption, and used to infect new culture flasks. Every forty passages in cell culture, the parasites were injected intraperitoneally into mice and were recovered from the peritoneal exudate three days after the infection. To prepare the inoculum, parasite suspensions were submitted to repeated passages through a 26-gauge needle until complete host cell disruption and centrifuged at low speed (100 × *g*/1 min at 4 °C) to remove host cell debris. The supernatant containing parasite suspension was collected and then washed twice (1000 × *g*/10 min at 4 °C) and the resulting pellet was resuspended in sterile PBS or RPMI-1640 for *in vivo* or *in vitro* experimental infections, respectively. Next, the parasite concentration was determined in a haemocytometer and was adjusted and viability confirmed by Trypan blue staining. Mice were inoculated by the intraperitoneal route (i.p.), with three different tachyzoite doses, as follows: a sub-lethal infection protocol (1.10^7^ tachyzoites/mice); a lethal infection to 50% of the mice (LD50; 2.10^7^ tachyzoites/mice), or a lethal infection to 100% of the mice (LD100; 3.10^7^ tachyzoites/mice). Immunological parameters (3–4 animals/group) were evaluated five days post-infection.

### Cell transfection and Immunofluorescence assay

Transfected monolayers of HEK-Peak cells (ATCC CRL-2828) were grown in RPMI medium containing 10% fetal bovine serum (FBS; Gibco-BRL). For all transfection studies, HEK293A were plated at 5.10^4^ cells/well in 24-well plates. After 24 h, cells were transfected with Nod2-HA plasmid (1 μg/well) by chemical transfection according to the manufacturer’s instructions (FuGENE 6, Roche, Mannheim, Germany). After 48 h, cells were infected with *N. caninum* tachyzoites and an immunofluorescence reaction was conducted 24 h post-infection, as previously described^37^. Briefly, wells were carefully washed with PBS and 4% formaldehyde/PBS was added to the wells for 20 min at room temperature. After new washing cycle with PBS, cells were incubated in a solution containing Triton X-100 at 1% and bovine serum albumin (BSA) at 2%, for 1 h at room temperature. Coverslips were then incubated for 1 h with a solution containing 0.5% Triton X-100, 0.5% BSA, primary policlonal antibody anti-HA (rabbit IgG; Sigma-Aldrich) and with hybridoma supernatant containing monoclonal antibodies against GRA2 protein (C3C5 clone). The cells were washed again and incubated with Alexa Fluor 594-conjugated anti-rabbit (Life Technologies) and FITC–conjugated anti-mouse secondary antibody (Sigma-Aldrich), for 1 h at room temperature. Finally, wells were washed with PBS, coverslips were inverted onto a glass slide with mounting solution containing 40,6-diamidino-2-phenylindole dihydrochloride solution (DAPI, Life Technologies) for nuclei staining. Images were obtained using inverted fluorescent microscope (EVOSfl, ThermoFisher Scientific, Waltham, MA, USA).

### Macrophage differentiation

Bone marrow-derived macrophages (BMDMs) were generated from WT and Nod2^−/−^ mice, as previously described^38^. Briefly, femurs were obtained from 6–8 week old mice, muscles were displaced using clean gauze and then both epiphyses were removed using sterile scissors and forceps. The bones were flushed with a syringe filled with RPMI-1640 to extrude bone marrow into a sterile polypropylene tube. The cell suspension generated was centrifuged at 500 × *g*, for 10 min, at 4 °C and resuspended in bone marrow differentiation media (R20/30), which is composed of RPMI1640 medium supplemented with 20% fetal bovine serum (Gibco-BRL), 30% L929-cell conditioned medium as a source of granulocyte/macrophage colony stimulating factor, 100 U/ml penicillin, 100 mg/ml streptomycin, and 2 mM L-glutamine. Cells were seeded in untreated sterile Optilux Petri dishes (BD Biosciences) and incubated at 37 °C in a 5% CO_2_ atmosphere. Four days after seeding the cells, an extra 10 ml of fresh R20/30 was added per plate and incubated for an additional 3 days. On day 7, the supernatants were discarded and the BMDMs were detached by gently pipetting ice-cold sterile PBS across the dish. The cells were centrifuged at 500 × *g*, for 10 min, at 4 °C, resuspended and analyzed by flow cytometry, and cell differentiation was followed by high expression of CD11b (over 95%). Once the positive phenotype was confirmed, BMDMs were plated at a concentration of 1.10^6^ cells/ml in RPMI-1640, supplemented with 10% FBS and exposed to *N. caninum* tachyzoites at means of infection (MOI) of 0.2 or 1 parasite/cell. Supernatants were collected after 8 and 24 h for cytokine measurements and cells were collected in TRIzol (Life Technologies) after 6 h for the evaluation of Nod2 transcripts.

### Western Blot Analysis

BMDMs were infected with *N. caninum* tachyzoites and were collected 48 hours post-infection in lysis buffer containing a mixture of protease and phosphatase inhibitors (Sigma-Aldrich). The expression of iNOS and Arginase I was evaluated by Western blot analyses, as previously described^39^. Briefly, protein concentration of the lysates were determined using a Bradford Protein Assay kit (Bio-Rad, Hercules, CA, USA). Proteins were separated using SDS polyacrylamide gel electrophoresis (30 μg/sample, SDS-PAGE- 8% for iNOS and 12% for Arginase I) and transferred onto nitrocellulose membranes (Life Technologies). The membranes were blocked with 5% dry milk (overnight) and incubated overnight at 4 °C with a sheep polyclonal antibody against murine Arginase I (1:500; R&D Systems, Minneapolis, MN, USA) or with a rabbit polyclonal antibody against murine iNOS (1:1,000; Sigma-Aldrich). The membranes were washed and incubated for 1 h at room temperature with HRP-conjugated secondary antibodies rabbit anti-sheep (1:10,000; Jackson Immunoresearch, Baltimore, USA) or with donkey polyclonal antibody anti-rabbit, respectively (1:10,000; GE Healthcare, Freiburg, Germany). A mouse monoclonal antibody against murine β-actin (1:10,000; Sigma-Aldrich) was used for loading controls in both membranes. The reactions were developed in an ECL solution (Bio-Rad Laboratories) and visualized through a dedicated imaging system (ChemiDocMP, Bio-Rad Laboratories). Densitometric data were normalized to the control (house-keeping) protein, using Scientific Imaging Systems (Image Lab 3.0 software; Bio-Rad Laboratories).

### Parasite proliferation assay

Parasites were collected, as previously described, washed twice (1000 × *g*, 10 min, 4 °C) and the resulting pellet was resuspended in sterile PBS. Next, parasites were quantified in a haemocytometer and diluted in sterile PBS in a mean concentration of 2 × 10^7^ tachyzoites/mL. Parasite suspensions obtained were pre-stained with 5 μM/mL of DDAO-SE (Life Technologies), for 10 min at 37 °C, according to previous description^40^. Tachyzoites were then washed with RPMI-1640 containing 10% FCS and, after further quantification in haemocytometer, they were used to infect BMDMs. After 24 h hours, the infected cells were harvested using EDTA (10 μM) solution, washed in PBS followed by centrifugation (500 × *g*/10 min at 4 °C), resuspended and read in a flow cytometer (FACSCantoII, BD Biosciences), in APC channel. A minimum of 50,000 events was acquired per sample. The collected data files were analyzed in FlowJo version 7.6.5 (Tree Star inc., Ashland, OR, USA).

### RNA extraction and real time quantitative PCR (qPCR)

RNA was extracted from WT and Nod2^−/−^ BMDMs by a combination of TRIzol (Life Technologies) protocol and an extraction Kit (Illustra RNA spin Mini, GE Healthcare), following manufacturers’ instructions. After extraction, RNA quantity, purity and quality were determined by a spectrophotometer (Thermo Scientific NanoDrop 1000). Complementary DNA (cDNA) was synthesized using 500 ng of RNA and reverse transcriptase SuperScript III (Life Technologies) on a regular thermocycler (PTC-100, MJ Research, Watertown, NY). The cDNA was amplified in a qPCR using SYBR Green (Life Technologies) and gene-specific primers in a Real-time PCR thermocycler (StepOne Plus, Life Technologies). The primers used for quantitative real-time RT-PCR amplification were as follow: *Gapdh* (forward 5′-ACCACAGTCCATGCCATCAC-3′, reverse 5′-TCCACCACCCTGTTGCTGTA-3′); *Hprt* (forward 5′-TGGAAAAGCCAAATACAAAGC-3′, reverse 5′-CAACATCAACAGGACTCCTCG-3′); *Nod2* (forward 5′-CGACATCTCCCACAGAGTTGTAATCC-3′, reverse 5′- GGCACCTGAAGTTGACATTTTGC-3′). Ct data (cycle threshold) were normalized to the expression of housekeeping gene (*Gapdh*) and analyzed using 2^−ΔΔCt^ method, where ΔΔCt = ΔCt sample –ΔCt control sample, in which ΔCt = Ct (target gene) –Ct (reference gene).

### DNA extraction and quantification of tissue parasite burden

Fragments of pancreas, lungs and livers were collected at day 5 post-infection (3–4 mice/group) and stored in a dry tube at −80 °C. To prepare the DNA samples, approximately 30 mg of tissue of each *N. caninum*-infected mouse or from 10^7^ *N. caninum* tachyzoites (Nc-1 isolate) were incubated in DNA extraction buffer (0.1 M Tris–HCl [pH 9.0], 1% SDS, 0.1 M NaCl, 1 mM EDTA) containing proteinase K (20 mg/mL) at 55 °C, overnight. Genomic DNA was extracted using a commercial kit (Kit Illustra Tissue & Cells Genomic Prep; GE Healthcare), following manufacturer’s instructions. DNA was quantified at 280 nm in a spectrophotometer (Thermo Scientific NanoDrop 1000) and parasite burden was assessed in 100 ng of DNA from each sample by quantitative PCR (qPCR) using SYBR Green (Life Technologies) intercalating reagent. The reaction was performed in a Real time PCR (StepOne Plus, Life Technologies) with the following parameters: 2 min at 50 °C, 10 min at 95 °C, forty cycles of 15 seconds (sec) at 95 °C, 30 sec at 58 °C and 30 sec at 72 °C, followed by a dissociation step, with temperature ranging from 60 to 95 °C. Amplification of parasite DNA was performed using primer pairs specific for *N. caninum* Nc5 gene (forward 5′-ACTGGAGGCACGCTGAACAC-3′, reverse 5′-GAAGGGGAGAAGCCAATTTC-3′). The results were obtained based on standard curve set with graded concentrations of parasite DNA.

### Histological analysis

Fragments of pancreas, lungs and liver of WT and Nod2^−/−^ mice were collected on 5 days after the infection (4 mice/group), fixed in 10% buffered formalin and included in paraffin. Tissue sections of 5 μm thickness were mounted on glass slides and histological assessment was performed after routine hematoxylin-eosin staining. The images were acquired with a digital camera (Leica DC300F, Leica Microsystems AG, Heerbrugg, Switzerland) coupled to a Leica DMR microscope (Leica Microsystems) and a computer, in a magnification of 200 and 400X, for histological analysis. The histological analysis was realized by a pathologist in a blind-fold manner, based on alterations in tissue morphology and cellularity, and the description was later matched with the identification of the slide by a code. These experiments were performed at least three times.

### Flow cytometry

Peritoneal exudates cells were harvested by washing the peritoneum of mice with 1 ml of cold sterile PBS, centrifuged at 500 × *g*, for 10 min, at 4 °C. Cells were then incubated for 30 minutes with a suspension containing normal rabbit serum (5%) in order to block unspecific Fc receptor binding. Next, antibodies anti-Ly6G/APC and anti-CD11b/FITC (BD Biosciences) were used (1:200) for surface antigen staining. The cells were washed and analyzed in a flow cytometer (FACSCantoII, BD Biosciences). A minimum of 100,000 events was acquired per sample and the collected data files were analyzed in FlowJo version 10.0 (Tree Star Inc.).

### Cytokine quantification

The concentration of IL-6, IL-10, TNF-α, in BMDM supernatants and peritoneal exudates were measured by commercial ELISA kits (BD Biosciences and R&D Systems), which were performed according to the manufacturers’ instructions. The reaction was revealed with peroxidase-streptavidin conjugate, followed by the addition of substrate containing hydrogen peroxide and TMB as a chromogen (BD Biosciences). Microplates were read in a plate reader (M2e, Molecular Devices, Sunnyvale, CA, USA) at 450 nm. Cytokine concentrations were calculated from standard curves of murine recombinant cytokines. The detection limits for the assays were 2,000 to 31.3 pg/mL.

### Measurement of MAPK phosphorylation

Peritoneal cells from WT and Nod2^−/−^ mice infected with *N. caninum* tachyzoites were collected and the preparation of samples was performed by a commercial kit, according to the manufacturer’s protocol for adherent cells (BD Biosciences). Briefly, denaturation buffer was added to a tube containing peritoneal cells and placed immediately in a boiling water bath for 5 min. Cell lysates were centrifuged at 14,000 × *g*, for 5 min, and supernatants were stored at –80 °C until measurement. pJNK1/2 (T183/Y185), pp38 (T180/Y182), and pERK1/2 (T202/Y204) were quantitatively determined using specific antibodies contained in the multiplex Flex Set Cytometric Bead Array kit (BD Biosciences). Acquisition and analysis was performed in a flow cytometer (FACSCantoII, BD Biosciences), and analyzed in dedicated software (FCAP array v3.0, BD Biosciences). The minimum detection levels for each phospho-protein was: pJNK = 0.38 U/ml; pp38 = 0.64 U/ml; pERK = 0.64 U/ml.

### Statistical analysis

Statistical significance of the results was checked using nonparametric one-way analysis of variance (ANOVA) followed by Bonferroni’s post-hoc test (for three or more groups) comparing all pairs of columns, or two-tailed Student’s t-test (for two groups). Differences in survival rates between groups were compared using Kaplan-Meier survival analysis, through a log-rank Mantel-Cox test. In all measurements, a P value under 0.05 (P < 0.05) was deemed as statistically significant. Analysis was performed with the aid of commercial software (GraphPad Prism, version 6.0, San Diego, CA, USA).

## Additional Information

**How to cite this article**: Davoli-Ferreira, M. *et al*. Nucleotide-binding oligomerization domain-containing protein 2 prompts potent inflammatory stimuli during *Neospora caninum* infection. *Sci. Rep.* **6**, 29289; doi: 10.1038/srep29289 (2016).

## Supplementary Material

Supplementary Information
