# Supplementary material for: Nucleotide-binding oligomerization domain-containing protein 2 prompts potent inflammatory stimuli during Neospora caninum infection
Source: Sci Rep. 2016 Jul 5;6:29289. doi: 10.1038/srep29289 (PMC4932631; doi:10.1038/srep29289)
Supplement: Supplementary Information [file srep29289-s1.pdf]

Nucleotide-binding oligomerization domain-containing protein 2 prompts potent inflammatory stimuli during *Neospora caninum* infection

Marcela Davoli-Ferreira[a,b], Denise M. Fonseca[b,d], Caroline M. Mota[a], Murilo S. Dias[b], Djalma S. Lima-Junior[b], Murilo V. da Silva[a], Gustavo F.S. Quirino[b], Dario S. Zamboni[c], João S. Silva[b], Tiago W.P. Mineo[a]*

Supplementary Figure 1.

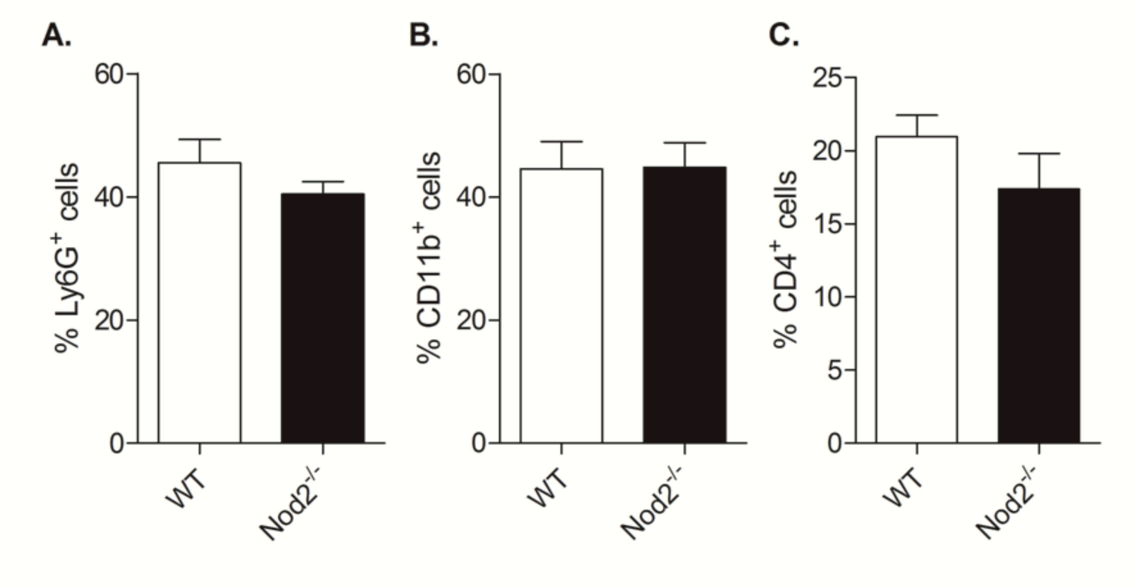

Peritoneal exudate cells from WT and Nod2$^{-/-}$ infected mice (LD100) were collect and the influx of Ly6G^{+} cells (A), CD11b^{+} cells (B) and CD4^{+} cells (C) was evaluated by flow cytometry, 5 days after infection.

Supplementary Figure 2.

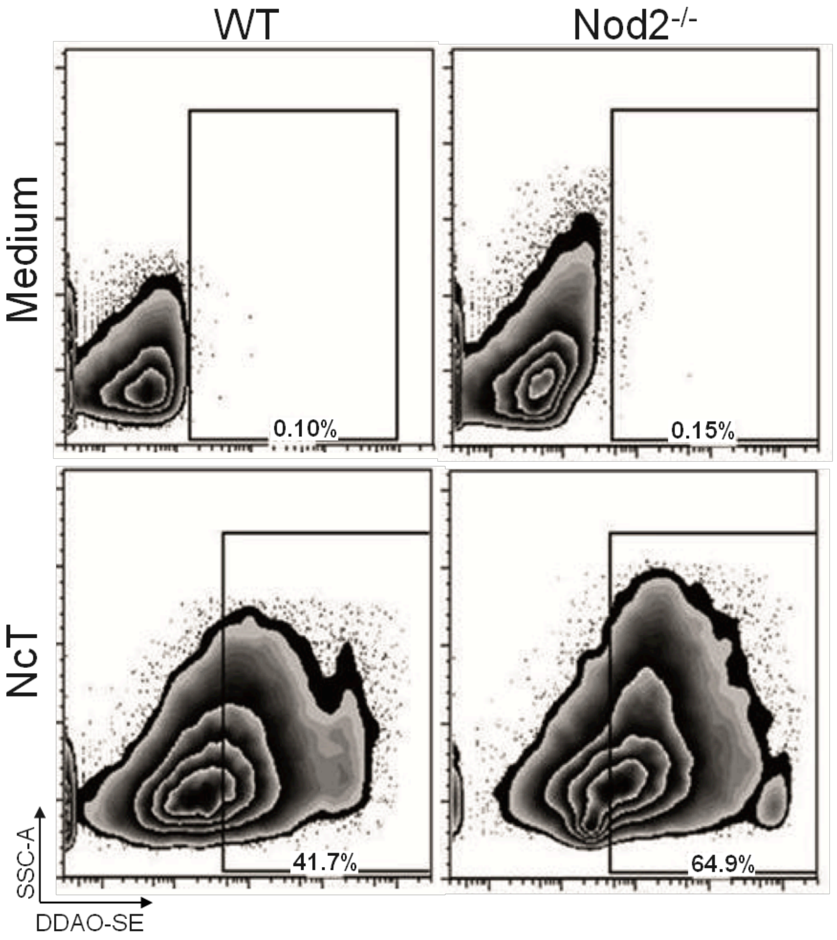

Representative dot plots of WT and Nod2$^{-/-}$ BMDMs infected with DDAO-stained *N. caninum* tachyzoites (MOI 0.2), 24 hours after infection. The collected data files were analyzed in FlowJo.

Supplementary Figure 3

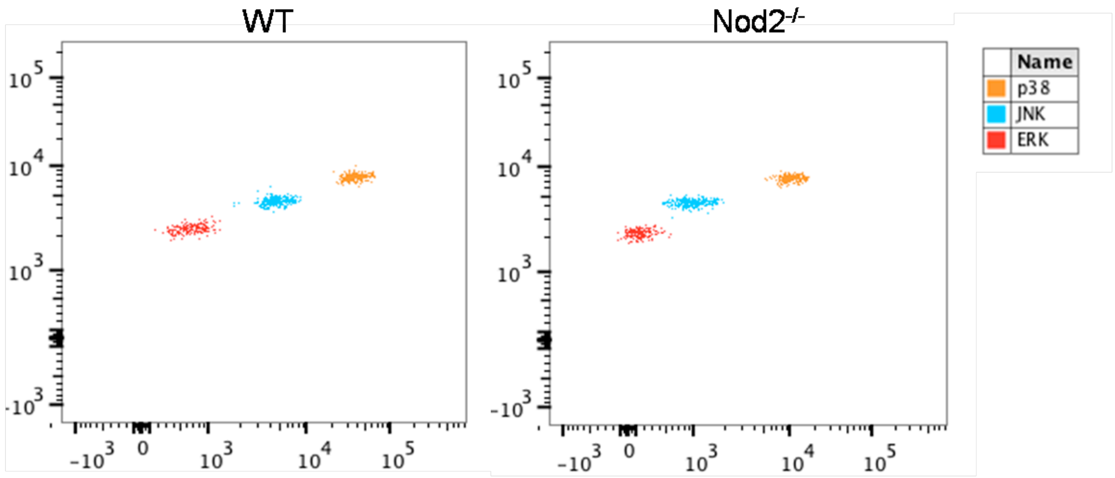

Representative dot plots of MAPK phosphorylation performed by CBA analysis of peritoneal exudate cells from Nod2$^{-/-}$ and WT mice, infected i.p. with 3.10^{7} *N. caninum* tachyzoites, 5 days post-infection.
